# Association between residence at birth and addiction service utilization: a spatial analysis of the Massachusetts birth record cohort

**DOI:** 10.3389/fepid.2025.1567257

**Published:** 2025-02-28

**Authors:** Yingjing Xia, Carolina Villanueva, Verónica M. Vieira

**Affiliations:** Joe C. Wen School of Population & Public Health, University of California, Irvine, Irvine, CA, United States

**Keywords:** epidemiology, substance use disorders, generalized additive models, addiction service utilization, spatial analysis

## Abstract

**Introduction:**

Substance use disorders impact a significant portion of the US population. Exposure to neighborhood environment early in life may contribute to disparities in policing, health outcomes and access to treatment for substance use disorders. Although many studies have examined the relationship between neighborhood context and substance use, few studies have accounted for the spatial distribution of substance use and social environment. The current study examined the association between birth address and substance addiction service utilization of individuals born in communities around the New Bedford Harbor Superfund site in southeast Massachusetts that face potential racial, socioeconomic, and environmental stressors.

**Methods:**

The analysis utilized birth record data between January 1992 and December 1998 (N = 12,151) from the Registry of Vital Records and Statistics with follow-up for substance addiction service utilization through June 2022 by the Bureau of Substances Addiction Services within the Massachusetts Department of Public Health (MADPH). We used generalized additive models (GAM) with a smooth for location to estimate local odds ratios (ORs) and 95% confidence intervals (CI) of substance addiction service utilization while adjusting for sociodemographic risk factors to identify important contributors to geographic disparities.

**Results:**

We found that birth addresses were significantly associated with substance addiction service utilization as a young adult (*p* = 0.037), with the highest statistically significant risk located closest to the harbor (OR = 1.42, 95% CI: 1.00, 2.02). Family education and prenatal care payer were significant predictors (*p* < 0.001) of substance addiction services use and strong spatial confounders.

**Discussion:**

The current study showed that significant associations between birth addresses and substance addiction service utilization later in life are primarily driven by socioeconomic predictors including family education and prenatal care payer.

## 1 Introduction

Substance use disorders impact a significant portion of the United States (US) population. A recent study estimated that 10% of the US population age 12 and above, 26.5 million people, meet the diagnostic criteria for substance use disorders (1). Among them, 4.6 million people have a severe substance use disorder (1). The prevalence of substance use disorders has a societal cost. For prescription opioid dependence and misuse alone, the total US cost was estimated at $55.7 billion in 2007, combining the effects on workplace, healthcare system and criminal justice system (2).

The prevalence of substance use disorders is similar across non-Hispanic White subpopulations (7.2%), Black subpopulations (9.2%), and Latino subpopulations (7.2%), yet disparities exist in the resulting consequences such as policing, health outcomes and access to treatment (3). For instance, the rates of arrest for possession and selling of any substance are higher for Black people than White people (4). Compared to non-Latino White adolescents with substance use disorders, Black adolescents reported receiving less specialty and informal care, and Latino adolescents reported receiving less informal care (5). Hispanic adolescents were less likely to have obtained alcohol-related treatment for their alcohol problems or dependence compared to non-Hispanic White adolescents, and more likely to report economics or logistic reasons for not being able to access care (6). Non-Hispanic Black persons and unhoused persons are overrepresented in opioid-related overdose deaths (7).

One possible contributing factor to these disparities is community characteristics. Molina et al. (8) found that for Asians and African Americans, living in affluent neighborhoods is associated with higher risk of substance use disorders compared to their non-Latino White counterparts, while for Latino residents, living in neighborhoods with high concentration of Latino residents is associated with lower risk of alcohol use disorders (8). A study examining treatment initiation and engagement in relation to community-level socioeconomic disadvantage and proportion of racial minority residents found that clients living in neighborhoods with high concentration of Black residents, Latino residents, or American Indian residents were less likely to initiate substance use treatment compared to their White counterparts (9). Furthermore, exposure to neighborhood environment early in life, especially during developmentally sensitive periods, can accumulate and affect substance use behavior later in adulthood (10, 11). Evidence shows that neighborhood characteristics in childhood can be associated with substance use behavior later in life. Lee et al. (12) found that childhood residential stability was negatively associated with problematic alcohol and marijuana use three decades later.

Although much work has been done on the relationship between community or neighborhood context and substance use, few studies have accounted for the spatial distribution of substance use and the environment. This is important because when spatial variation of a risk factor is similar to the disease outcome, location itself may be confounding (13). Spatial confounding can result in biased estimates for the relationship between risk factors and outcome (14). A study mapping the spatial distributions of socioeconomic vulnerability, physical environment and substance use treatment outcomes in Buffalo, NY demonstrated that the area with the highest risk for negative treatment outcomes are in areas of high-risk in socioeconomic disadvantage and physical environment, which suggests potential spatial confounding (15).

The goal of the current study is two-fold. First, we examined the association between birth address and future substance addiction service utilization of individuals born between 1992 and 1998 in four towns near the Massachusetts Superfund site (i.e., Acushnet, Dartmouth, Fairhaven, and New Bedford). Second, we examined the relationship between residential addresses at birth and substance addiction service use while adjusting for sociodemographic risk factors to identify any potential spatial confounding. If these sociodemographic factors do not account for any geographic variation observed, additional factors should be considered. The study region was chosen due to its proximity to the New Bedford Harboe Superfund site, which is highly contaminated with polychlorinated biphenyls (PCBs). Dredging of the Harbor's sediments, which began in 1994 and continued for almost three decades, potentially exposed residents to PCBs. Previous studies have shown that exposure to PCBs is associated with behavioral outcomes, including teen birth and risk-taking related behavior, potentially through impaired development of the prefrontal cortex, which is critical to executive function but is not yet fully developed in adolescence (16, 17). In addition to environmental stressors, the residents surrounding the NBH also experience economic and social stressors, with 22% of New Bedford residents living below the poverty level, many of whom are non-English speaking immigrants from Cape Verde and Portugal (17). Therefore, this region offers a unique opportunity to examine how location at birth, a proxy for social and community stressors, is associated with substance use behavior later in life.

## 2 Methods and materials

### 2.1 Study population

**Figure 1:**
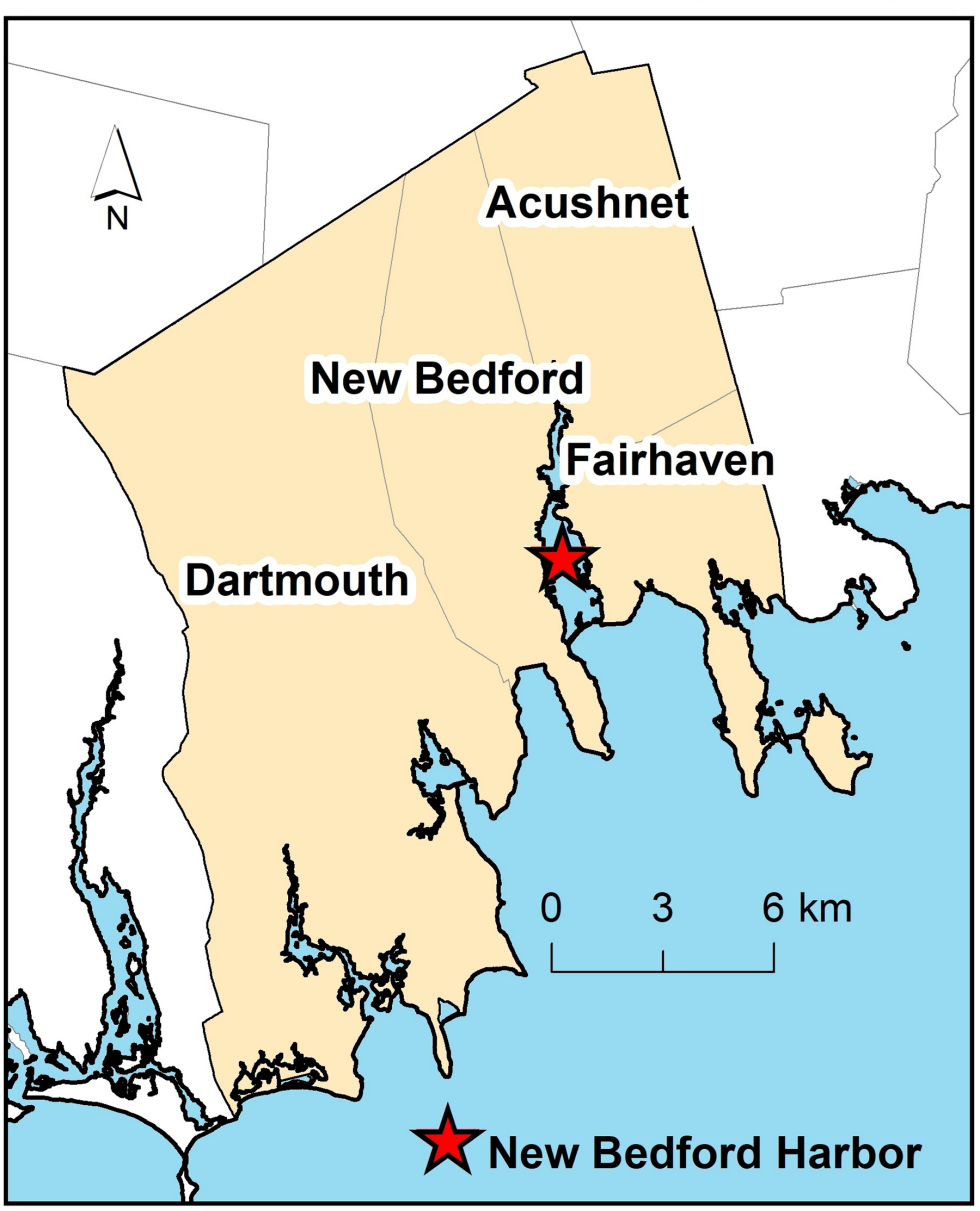
The study area consists of four southeastern Massachusetts towns (New Bedford, Acushnet, Dartmouth, and Fairhaven) surrounding the New Bedford harbor superfund site (represented with a red star).

The current analysis utilized birth record data from the Registry of Vital Records and Statistics within the Massachusetts Department of Public Health (MADPH). The MA Birth Record Cohort (MABRC) consisted of children born in Acushnet, Dartmouth, Fairhaven, and New Bedford towns between January 1992 and December 1998 (*N* = 12,151) (17). Figure 1 shows the location of the study towns in relation to the New Bedford Harbor Superfund site. The MABRC includes information on parent demographic (maternal age, marital status, maternal and paternal occupation, and education years), pregnancy (pregnancy weight gain, gestational age, prenatal exposure to tobacco or alcohol, birthweight, breastfeeding, parity), and prenatal care (adequacy, prenatal care and delivery source of payer).

### 2.2 Outcome

Information on the use of substance addiction services was provided by the Bureau of Substances Addiction Services (BSAS) within MADPH. Among its array of services, the BSAS oversees licensing for substance use disorders treatment programs in Massachusetts and monitors treatment use statewide. BSAS services (18) are provided to Massachusetts residents of age 12 years and older. Since children in the MABRC were born between 1992 and 1998, we included BSAS service data from 2004 and onward for the linkage of birth records to BSAS services. Children in the MABRC who received at least one BSAS service between 2004 and June 30, 2022, the time when data was last updated, were defined as having used substance addiction services.

### 2.3 Covariates

The covariates adjusted in the primary analysis were determined *a priori* based on study design and confounding. These covariates included family education, prenatal care payer, maternal marital status (married, not married), maternal age, maternal race (Black mother, White mother, other race), maternal ethnicity (Hispanic woman, non-Hispanic woman), child's sex (male, female), and birth year. Family education was characterized by the highest education obtained between mothers and fathers (if reported) and categorized into 5 levels: less than high school, high school/GED, some college, college, and graduate/advanced degrees. Prenatal care payer was categorized into three levels: public, private, and other. If prenatal care payer was not available, we used the delivery payer if available. Education and payer information were proxies for individual-level socio-economic status.

### 2.4 Statistical methods

Local odds ratios (ORs) of substance addiction service use across the study area were estimated with generalized additive models (GAM) (19–23), a semi-parametric or nonparametric model. Birth addresses were modeled with a bivariate smooth function (*S*) of latitude (*x*_1_) and longitude (*x*_2_)

$$\mathrm{logit}[\;p(x_{1},x_{2})]=S(x_{1},x_{2})+\gamma^{\prime }z$$

where the left side is the log of odds ratio of substance addiction service use at location (*x*_1,_ *x*_2_), *S* is a loess smooth function, ***z*** is a vector of covariates, and *γ* is a vector of parameters. The degree of smoothing is dependent on the span of the loess function which represents the portion of the data used in the locally weighted smoothing. Optimal span size for the smooth function was determined by minimizing the Akaike's information Criterion (AIC). The loess smooth adapts to changes in population density while allowing for adjustment of individual-level covariates to identify local areas of risk near the NBH. The advantage of this approach is that data are not aggregated at a town level, allowing for targeted intervention.

The global null hypothesis is that the odds of substance addiction service use does not depend on the location of birth. We calculated a permutation-based *p*-value (24). For each permutation test, we randomly reassigned locations to subjects and calculated the deviance statistics using the optimal span size for the observed model. We repeated this permutation procedure for 999 times and divided the rank of the observed value by 1,000 to obtain an approximate permutation *p*-value. Local pointwise standard errors and confidence intervals were used to calculate areas of statistical significance (22).

We created a grid covering the study area using the minimum and maximum of the longitude and latitude coordinates. For each GAM model, we computed predicted log odds of substance addiction service use at each grid point for an individual with median value of the continuous covariates and reference level for categorical covariates and used the median of the predictions to calculate ORs. We mapped the predicted values using dark blue to dark red continuous color scale and a common range of ORs. We mapped the predicted ORs using the optimal span size identified by the AIC and a common span size for comparison across maps. Black contour lines denote areas of statistical significance.

We started by fitting a crude model with only the smooth function and without adjusting for any covariates to examine the spatial distribution of addiction service use. Then, we fit a fully adjusted model and a set of stepwise models to examine spatial confounding conditioning on other covariates. The order of the stepwise models was determined by the strength of univariate associations between each individual covariate and the outcome in logistic regressions. Lastly, we fit univariable GAM models for each covariate to examine how the individual covariate contributed to the spatial variation of substance addiction service use.

Analyses and mapping were conducted in R (v4.4.2) using MapGAM package (25–29). The study was approved by the ethics institutional review boards at University of California Irvine IRB and the MADPH.

## 3 Results

### 3.1 Demographics of MA birth record cohort

Out of 12,151 children born between 1992 and 1998 in the study area, 5,852 of them were assigned female at birth (48.2%). The majority of them were born to mothers who identified as a White mother (79.4%), followed by Other race (16.0%) and Black race (4.6%). Approximately half (*n* = 6,094, 50.1%) used a private source of prenatal payment and another 5,488 (45.2%) used a public source. Compared to children who did not subsequently utilize substance addiction services, the ones who did were more likely to be assigned male at birth and have a family education of high school or less ($\chi_{3}^{2}=98.925$, *p* < 0.0001). Mothers of children who subsequently used addiction services were younger on average [service use: Mean (SD) = 24.6 (5.7); no service use: Mean (SD) = 26.0 (5.9), $\chi_{1}^{2}=35.87$, *p* < 0.001], more likely to have used public insurance to pay for prenatal care ($\chi_{2}^{2}=52.289$, *p* < 0.001), and more likely to be not married ($\chi_{1}^{2}=43.863$, *p* < 0.001) than those who did not use addiction services. Detailed demographic information and univariate logistic regression results are presented in Table 1.

**Table 1:** Demographic characteristics of MA Birth Record Cohort by substance abuse service utilization.

Characteristics	MA birth record cohort by utilization status			
	No (*N* = 11,552)	Yes (*N* = 599)	Total (*N* = 12,151)	*p* value
Family education				< 0.001
Less than high school	2,012 (17.4%)	163 (27.2%)	2,175 (17.8%)	
High school	3,677 (31.8%)	224 (37.4%)	3,901 (32.1%)	
Some college associate degree	2,819 (24.4%)	144 (24.0%)	2,963 (24.4%)	
College degree	1,304 (11.2%)	24 (4.0%)	1,328 (10.9%)	
Graduate degree	727 (6.3%)	9 (1.5%)	736 (6.1%)	
Missing	1,013 (8.8%)	35 (5.8%)	1,048 (8.6%)	
Prenatal care payer				<0.001
Private	5,879 (50.8%)	215 (35.9%)	6,094 (50.1%)	
Public	5,130 (44.4%)	358 (59.8%)	5,488 (45.2%)	
Other	496 (4.3%)	23 (3.8%)	519 (4.3%)	
Missing	47 (0.4%)	3 (0.5%)	50 (0.4%)	
Maternal marital status				<0.001
Married	6,737 (58.3%)	266 (44.4%)	7,003 (57.6%)	
Not married	4,789 (41.5%)	332 (55.4%)	5,121 (42.1%)	
Missing	26 (0.2%)	1 (0.2%)	27 (0.2%)	
Maternal age				<0.001
Mean years (SD)	26.0 (5.9)	24.6 (5.7)	25.9 (5.9)	
Range	13.0- 47.0	14.0–42.0	13.0–47.0	
Maternal race				0.19
White mother	9,151 (79.2%)	472 (78.8%)	9,623 (79.2%)	
Black mother	521 (4.5%)	36 (6.0%)	557 (4.6%)	
Other race	1,855 (16.1%)	89 (14.9%)	1,944 (16.0%)	
Missing race	25 (0.2%)	2 (0.3%)	27 (0.2%)	
Infant sex at birth (female)	5,657 (49.0%)	195 (32.6%)	5,852 (48.2%)	<0.001
Ethnicity (Hispanic)	1,191 (10.3%)	60 (10.0%)	1,251 (10.3%)	0.818
Birth year				<0.001
1992	1,750 (15.1%)	163 (27.2%)	1,913 (15.7%)	
1993	1,689 (14.6%)	118 (19.7%)	1,807 (14.9%)	
1994	1,682 (14.6%)	114 (19.0%)	1,796 (14.8%)	
1995	1,537 (13.3%)	77 (12.9%)	1,614 (13.3%)	
1996	1,569 (13.6%)	61 (10.2%)	1,630 (13.4%)	
1997	1,590 (13.8%)	40 (6.7%)	1,630 (13.4%)	
1998	1,735 (15.0%)	26 (4.3%)	1,761 (14.5%)	

For continuous variables, *p* values are calculated from analysis of variance (ANOVA) tests. For categorical variables, *p* values are calculated based on is $\chi^{2}$ tests.

In fully-adjusted models, several maternal sociodemographic variables were associated with greater odds of substance use later in life. Children of mothers who used public prenatal insurance [OR, 1.40: 95% Confidence Interval (CI), 1.12–1.75 compared to those with private insurance] and mothers who were not married (OR, 1.28: 95% CI, 1.03–1.59 compared to those who were married) had higher odds of addiction service use in later life while each increasing level of maternal education was protective (Table 2, last column). There were no significant differences in addiction service use by maternal race or ethnicity.

**Table 2:** Estimates of covariates, optimal span size, global *p*-values, and predicted OR range for each step-wise GAM model.

Model output	Model covariates		Step-wise GAM models								
			Crude	Step 1	Step 2	Step 3	Step 4	Step 5	Step 6	Step 7	Fully adjusted
Optimal span			0.65	0.65	0.8	0.8	0.95	0.95	0.95	0.95	0.95
Global *P*-value			0.04	0.09	0.26	0.33	0.26	0.19	0.18	0.18	0.28
Predicted range			0.64–1.56	0.61–1.45	0.82–2.34	0.81–2.26	0.85–2.06	0.84–2.14	0.84–2.14	0.85–2.04	0.85–2.04
Odds ratio [95% CI]	Year			0.77 [0.74, 0.81]	0.77 [0.74, 0.81]	0.77 [0.74, 0.81]	0.77 [0.73, 0.81]	0.77 [0.73, 0.80]	0.77 [0.73, 0.80]	0.77 [0.73, 0.80]	0.77 [0.73, 0.80]
	Family education (ref: less than high school)	High school			0.78 [0.63, 0.96]	0.79 [0.64, 0.97]	0.84 [0.68, 1.05]	0.86 [0.70, 1.07]	0.87 [0.70, 1.08]	0.84 [0.67, 1.05]	0.83 [0.67, 1.04]
		Some College/Associate degree			0.69 [0.55, 0.88]	0.69 [0.55, 0.87]	0.79 [0.62, 1.01]	0.82 [0.64, 1.05]	0.83 [0.64, 1.07]	0.80 [0.62, 1.03]	0.79 [0.62, 1.02]
		College			0.25 [0.16, 0.38]	0.25 [0.16, 0.38]	0.31 [0.20, 0.50]	0.34 [0.21, 0.54]	0.35 [0.22, 0. 55]	0.33 [0.21, 0.53]	0.33 [0.21, 0.53]
		Graduate Degree			0.16 [0.08, 0.32]	0.16 [0.08, 0.32]	0.21 [0.11, 0.43]	0.23 [0.11, 0.46]	0.23 [0.12, 0.47]	0.23 [0.11, 0.45]	0.22 [0.11, 0.45]
	Infant sex(ref: male)	Female				0.52 [0.44, 0.63]	0.52 [0.43, 0.62]	0.52 [0.43, 0.62]	0.52 [0.43, 0.62]	0.52 [0.44, 0.63]	0.52 [0.44, 0.63]
	Prenatal care payer (ref: private)	Public					1.51 [1.24, 1.83]	1.34 [1.07, 1.67]	1.33 [1.06, 1.66]	1.39 [1.11, 1.74]	1.40 [1.12, 1.75]
		Other					1.18 [0.76, 1.85]	1.13 [0.72, 1.77]	1.12 [0.72, 1.77]	1.14 [0.72, 1.79]	1.14 [0.72, 1.79]
	Maternal marriage Status(ref: married)	Not Married						1.27 [1.03, 1.56]	1.25 [1.01, 1.55]	1.28 [1.03, 1.60]	1.28 [1.03, 1.59]
	Maternal age								1.00 [0.98, 1.01]	1.00 [0.98, 1.01]	1.00 [0.98, 1.01]
	Maternal race (ref: White)	Black								1.05 [0.72, 1.53]	1.05 [0.72, 1.53]
		Other								0.69 [0.54, 0.89]	0.73 [0.52, 1.03]
	Ethnicity (ref: non- Hispanic)	Hispanic									0.91 [0.61, 1.36]

### 3.2 Generalized additive models

**Figure 2:**
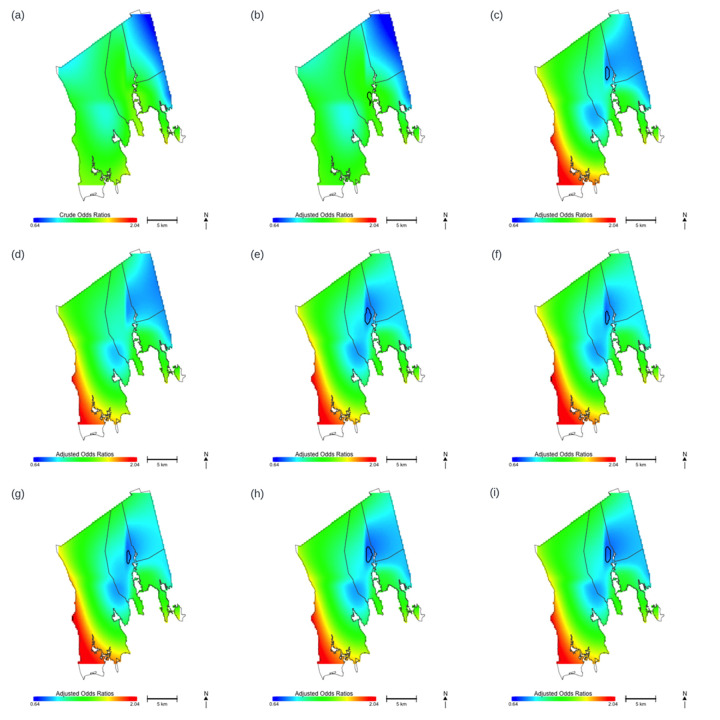
Maps of predicted odds ratios for substance abuse services utilization by location, using a common span size (0.65) and a common range (0.64–2.04). Areas in blue are where risk is lowest and areas in red are where risk is highest. Black contour lines denote statistically significant areas in New Bedford, north of the harbor, in models including family education and prenatal care payer. **(a)** Crude model without adjusting for any covariates. **(b)** The model adjusted for birth year. **(c)** The model adjusted for birth year and family education. **(d)** The model adjusted for birth year, family education and infant sex. **(e)** The model adjusted for birth year, family education, infant sex and prenatal care payer. **(f)** The model adjusted for birth year, family education, infant sex, prenatal care payer, and maternal marital status. **(g)** The model adjusted for birth year, family education, infant sex, prenatal care payer, maternal marital status, and maternal age. **(h)** The model adjusted for birth year, family education, infant sex, prenatal care payer, maternal marital status, maternal age and maternal race. **(i)** The model adjusted for birth year, family education, infant sex, prenatal care payer, maternal marital status, maternal age, maternal race and ethnicity.

Before adjusting for any covariates, birth addresses were significantly associated with addiction service use (permutation *p* = 0.037). The map of predicted ORs showed lower predicted ORs of addiction service use in the eastern part of Acushnet and Fairhaven (Figure 2a). The highest statistically significant risk was located closest to the harbor (OR = 1.42, 95% CI: 1.00, 2.02) Once fully adjusted for covariates, birth addresses were no longer significantly associated with substance addiction service use (permutation *p*-value = 0.28).

We conducted stepwise models to examine spatial confounding of each covariate. The order of the covariates was as follows: birth year, family education, infant sex, prenatal care payer, maternal marital status, maternal age, maternal race and ethnicity. This order was determined by the strength of univariate association between the covariate and addiction service use. The resulting maps of predicted ORs modeled with a common span size of 0.65 are displayed in Figures 2b–i. Optimal span size, permutation *p*-values, predicted OR range, and estimates for each covariate are presented in Table 2.

Comparing each map to the one from the step prior, adjusting for family education noticeably changed the appearance of the map, while birth year, infant sex, prenatal payer, maternal marital status, maternal age, and maternal race did not. The fully adjusted map showed significantly low ORs of substance addiction service use on the east side of New Bedford, close to Acushnet, denoted by the black contour in the Figure 2i and greater ORs, although not significant, in the lower west side of Dartmouth.

**Figure 3:**
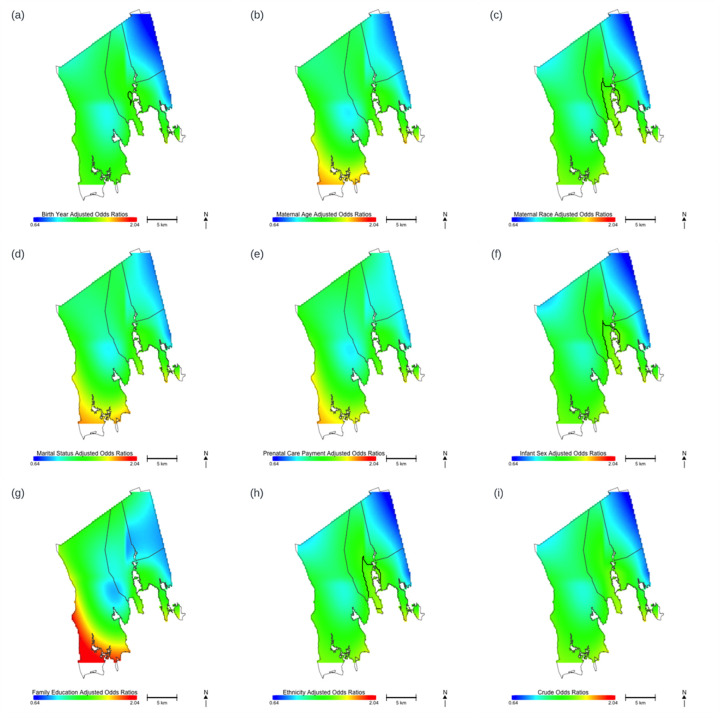
Maps of predicted odds ratios for substance abuse services utilization by location, using a common span size (0.65) and a common range (0.64–2.04), adjusting for one covariate at a time. Areas in blue are where risk is lowest and areas in red are where risk is highest. Map displays the predicted ORs for substance addiction services utilization associated with location for. **(a)** The model adjusted for birth year. **(b)** The model adjusted for maternal age. **(c)** The model adjusted for maternal race. **(d)** The model adjusted for maternal marital status. **(e)** The model adjusted for prenatal care payer. **(f)** The model adjusted for infant sex. **(g)** The model adjusted for family education. **(h)** The model adjusted for ethnicity. **(i)** Crude model without adjusting for any covariates.

To verify the observation from stepwise models, we fit GAM models adjusting for each covariate one at a time (Figure 3, Table 3). These univariate models confirmed that family education changed appearance of the predicted odds ratio the most, followed by prenatal care payer, maternal age, and marital status. The results of the univariate models support the stepwise models, where evidence suggests spatial confounding of family education and prenatal care payer in association with substance addiction service use.

**Table 3:** Estimates of covariates, optimal span size, global *p*-values, and predicted OR range for single covariate GAM models.

Model output	Model covariates		Single covariate GAM models						
			Family education	Infant sex	Prenatal care payer	Maternal marriage status	Maternal age	Maternal race	Ethnicity
Optimal span			0.80	0.65	0.75	0.75	0.70	0.65	0.65
Global *P*-value			0.19	0.02	0.45	0.24	0.20	0.03	0.02
Predicted range	(Crude model: 0.64–1.56)		0.84–2.43	0.63 - 1.51	0.85–1.73	0.78–1.72	0.73–1.72	0.64–1.54	0.63–1.55
Odds ratio [95% CI	Family education	Less than high school	1.00 ref						
		High school	0.75 [0.61, 0.93]						
		Some college associate degree	0.63 [0.50, 0.79]						
		College	0.22 [0.14, 0.34]						
		Graduate degree	0.14 [0.07, 0.29]						
	Infant sex	Male		1.00 ref					
		Female		0.50 [0.42, 0.60]					
	Prenatal care payer	Private			1.00 ref				
		Public			1.90 [1.59, 2.26]				
		Other			1.26 [0.81, 1.95]				
	Maternal Marriage status	Married				1.00 ref			
		Not married				1.74 [1.47, 2.05]			
	Maternal age						0.96 [0.94, 0.97]		
	Maternal race	White						1.00 ref	
		Black						1.26 [0.89, 1.79]	
		Other						0.88 [0.70, 1.11]	
	Ethnicity	Non-Hispanic							1.00 ref
		Hispanic							0.91 [0.69, 1.20]

## 4 Discussion

Birth address is a proxy measure of one's environment at birth, which can include the built environment, social interactions and networks, and access to local services and institutions (30). Therefore, the association between birth addresses and addiction service utilization later in life may reflect early exposure to sociodemographic risk factors that subsequently impact substance use behavior and treatment. Our findings show that individual level socioeconomic factors, such as family education and prenatal care payer, largely account for the spatial variation of birth address and addiction service utilization. Factors that are associated with both location and substance addiction service utilization are considered spatial confounders. Family education may affect substance addiction service utilization directly via health literacy and indirectly as a correlate of income. Substance addiction service utilization is associated with forms of payment accepted and the types of travels needed to access the treatment. A recent study found that the most accepted form of payment at substance use disorders treatment facility was cash, followed by private insurance and then public insurance (31). Therefore, patients with more financial resources and private insurance are likely to have more choices in treatment facilities and easier access to them. Additionally, substance use disorder treatment facilities accepting Medicaid in the US are also less likely to be in counties that have a higher percentage of minority, rural, or uninsured residents (32). Patients with public insurance or uninsured, and lower socioeconomic status may need to travel further to access a treatment facility and have less choices due to their insurance, which may discourage treatment utilization (33).

Additionally, exposure to socioeconomic hardship early in life can be a risk factor for substance use in adolescence and adulthood. It is hypothesized that children from more socioeconomically disadvantaged environment may also be exposed to worse physical environment in addition to lack of financial resources. These stressors can accumulate and elevate the risk for substance use later in life (34). Prospective studies have shown that childhood socioeconomic status is associated with drug use later in life, even after controlling for psychiatric diagnoses and parental psychiatric histories (35, 36). Therefore, it is also possible that exposure to socioeconomic factors at birth contributes to risk of substance use later in life and therefore accounts for the spatial variation between birth addresses and addiction service utilization.

Prior studies have found spatial variation in outcomes such as substance use and locations of addiction treatment services. Using health record data from a large urban care center in North Carolina, Cobert et al. (37) found that residential address of patients admitted for misuse of illicit drugs and overdoses showed spatial heterogeneity, which unlike our study, remained even after adjusting for covariates including area deprivation. Similarly, another study on opioid overdose deaths in Flint and Genesee County, Michigan, observed geospatial clusters (38) particularly in poorer areas. In addition, locations of treatment facilities for substance use also show spatial variation. Spatial analysis on accessibility of medication for opioid use disorder across US census tracts showed large gaps of “treatment deserts” that disproportionately impacted rural areas (39). It is possible that the association between birth addresses and addiction service utilization later in life reflects spatial variation of substance use and availability of treatment facilities.

There are a few limitations to our study. First, we do not have information on the type or severity of the substance use behavior that led to addiction service utilization. Relationship between birth address and addiction service utilization may differ based on the type of substances and the severity of substance use disorders. Second, we do not have information on location of treatment facilities or timing of treatment and or subsequent engagement in services, which may also vary by sociodemographic factors and neighborhood (9). Furthermore, we do not have information on the age at which treatment service was utilized. Substance use early in life may be affected more by exposure at birth compared to substance use developed later in life. However, the cohort that we examined was born between 1992 and 1998, so the services were mostly accessed during adolescence to early adulthood. Additionally, we were limited to socioeconomic characteristics available in the birth records. There is the potential for unmeasured confounding due to home environment (e.g., relationship with parents, homelessness, foster care), cultural or familial patterns of drinking, and poor school performance or dropout. While we used prenatal care payment source as a proxy for socioeconomic status, there may be residual confounding due to unmeasured individual household income data. Results may also reflect other important geographic variables such as neighborhood safety or access to community health clinics.

The current study also has several strengths. We used a prospective cohort that linked birth record data with later substance addiction service use and thereby avoided potential recall bias. Individuals included in the MA Birth Record Cohort resided in proximity to PCB contaminated New Bedford Superfund Site and may be at higher risk for behavior outcomes such as substance use. We used generalized additive models with a smooth for location to predict risk for a spatially continuous study area and were able to adjust for important family characteristics. Our findings show that addiction service utilization in this region varies spatially by birth address and socioeconomic factors were contributing factors of this spatial variation.

The knowledge that sociodemographic risk factors such as family education and prenatal care payer explains the geographic disparities that we observed provides actionable insights for targeted interventions. By identifying vulnerable populations based on their neighborhood and individual characteristics, local policy makers and health departments can strategically enhance the availability of substance abuse risk-reduction and health literacy interventions for those who might benefit most.

## 5 Conclusion

The current study examined whether birth addresses were associated with substance addiction service utilization later in life, and whether sociodemographic risk factors spatially confounded the relationship between birth addresses and addiction service utilization using data from Massachusetts Birth Record Cohort. We found that birth addresses were significantly associated with substance addiction service utilization later in life. Once we adjusted for socioeconomic predictors such as family education and prenatal care payer, we no longer saw a significant association between address at birth and addiction service use later in life.

## Data Availability

The data analyzed in this study is subject to the following licenses/restrictions: Data can only be provided by the Registry of Vital Records and Statistics within the Massachusetts Department of Public Health. The code can be obtained by contacting the corresponding author.
